# Quality of life among Iranian patients with beta-thalassemia major using the SF-36 questionnaire

**DOI:** 10.1590/1516-3180.2013.1313470

**Published:** 2013-06-01

**Authors:** Sezaneh Haghpanah, Shiva Nasirabadi, Fariborz Ghaffarpasand, Rahmatollah Karami, Mojtaba Mahmoodi, Shirin Parand, Mehran Karimi

**Affiliations:** I MD, MPH. Assistant Professor and Internal Manager, Hematology Research Center, Shiraz University of Medical Sciences, Shiraz, Iran.; II BSc. Master’s Student and Genetics Specialist, Hematology Research Center, Shiraz University of Medical Sciences, Shiraz, Iran.; III MD. Physician, Health Policy Research Center, Shiraz University of Medical Sciences, Shiraz, Iran.; IV MA. Assistant Editor, Hematology Research Center, Shiraz University of Medical Sciences, Shiraz, Iran.; V MD. Professor of Pediatric Hematology and Oncology, Hematology Research Center, Shiraz University of Medical Sciences, Shiraz, Iran.

**Keywords:** Beta-thalassemia, Chronic disease, Health status, Mental health, Quality of life, Talassemia beta, Doença crônica, Nível de saúde, Saúde mental, Qualidade de vida

## Abstract

**CONTEXT AND OBJECTIVE:**

Patients with beta-thalassemia major (β-TM) experience physical, psychological and social problems that lead to decreased quality of life (QoL). The aim here was to measure health-related QoL and its determinants among patients with β-TM, using the Short Form-36 (SF-36) questionnaire.

**DESIGN AND SETTING:**

Cross-sectional study at the Hematology Research Center of Shiraz University of Medical Sciences, in southern Iran.

**METHODS:**

One hundred and one patients with β-TM were randomly selected. After the participants’ demographics and disease characteristics had been recorded, they were asked to fill out the SF-36 questionnaire. The correlations of clinical and demographic factors with the QoL score were evaluated.

**RESULTS:**

There were 44 men and 57 women of mean age 19.52 ± 4.3 years (range 12-38). On two scales, pain (P = 0.041) and emotional role (P = 0.009), the women showed significantly lower scores than the men. Lower income, poor compliance with iron-chelating therapy and presence of comorbidities were significantly correlated with lower SF-36 scores. These factors were also found to be determinants of worse SF-36 scores in multivariate analysis.

**CONCLUSIONS:**

We showed that the presence of disease complications, poor compliance with iron-chelating therapy and poor economic status were predictors of worse QoL among patients with β-TM. Prevention and proper management of disease-related complications, increased knowledge among patients regarding the importance of managing comorbidities and greater compliance with iron-chelating therapy, along with psychosocial and financial support, could help these patients to cope better with this chronic disease state.

## RESUMO

**CONTEXTO E OBJETIVO:**

Pacientes com beta-talassemia maior (β-TM) vivenciam problemas físicos, psicológicos e sociais que levam à diminuição da qualidade de vida (QV). O objetivo foi determinar a QV relacionada à saúde e seus determinantes em pacientes com β-TM, utilizando questionário SF-36 (Short Form-36).

**TIPO DE ESTUDO E LOCAL:**

Estudo transversal no Centro de Hematologia e Pesquisa em Universidade de Ciências Médicas de Shiraz, no sul do Irã.

**MÉTODOS:**

Foram selecionados aleatoriamente 101 pacientes com β-TM. Após registro demográfico e características da doença, eles foram convidados a preencher o questionário SF-36. A correlação entre fatores clínicos e demográficos com escore de QV foi avaliada.

**RESULTADOS:**

Havia 44 homens e 57 mulheres, com idade média de 19,52 ± 4,3 (variação 12-38) anos. Em duas escalas, dor (P = 0,041) e aspectos emocionais (P = 0,009), as mulheres apresentaram escores significativamente menores aos dos homens. Menor renda, baixa adesão à terapia quelante de ferro e presença de comorbidades foram correlacionadas com escores SF-36 significativamente menores. Esses fatores foram também considerados determinantes de piores escores de SF-36 em análise multivariada.

**CONCLUSÕES:**

Mostramos que a presença de complicações da doença, a baixa adesão ao tratamento da terapia quelante de ferro e o baixo status econômico são preditores de pior QV em pacientes com β-TM. Prevenção e manejo adequado das complicações relacionadas com a doença, aumento do conhecimento dos pacientes sobre a importância do gerenciamento de comorbidades e ter maior adesão ao tratamento quelante de ferro, considerando também o apoio psicossocial e financeiro, poderiam ser úteis para melhor lidar com esse estado de doença crônica.

## INTRODUCTION

Beta-thalassemia major (b-TM), also known as Cooley’s anemia, is a hemoglobinopathy leading to chronic hemolytic anemia. In individuals with b-TM, there is either complete absence of b-globin gene production or a partial reduction. If patients with b-TM are treated inadequately and inappropriately, it will be a fatal disease.^1^ It is a common disease in Mediterranean countries, Southeast Asia, the Indian subcontinent and the Middle East, including Iran.^2^^,^^3^

The life expectancy and survival of these patients have increased dramatically over previous decades through introduction of regular blood transfusion therapy and iron-chelating therapies.^4^^,^^5^^,^^6^ As well as these patients’ survival, their quality of life (QoL) is believed to be lower than that of the normal population because of a variety of issues that these patients encounter during their lives, including the presence of comorbid chronic conditions, treatment components such as periodic regular hospital visits for regular transfusions and painful injections, appearance, absence of sexual development, infertility, inability to raise their own family, disease complications, uncertainties about the future, psychiatric disorders, and difficulties in employment and playing a role within society.^7^^,^^8^ Because of these factors, patients with b-TM experience many physical, psychological and social problems that lead to decreased QoL. Culture and education are two independent factors that influence the QoL of patients with b-TM to a great extent.^9^

SF-36 is a short-form health survey including 36 questions. It is a well-recognized, self-administered and user-friendly questionnaire for measuring QoL in general populations as well as in populations with specific conditions.^10^ The validity and reliability of the Persian-translated version of this instrument for measuring QoL among thalassemic patients was approved previously by Jafari et al.^11^ Information on these patients’ QoL can help healthcare providers to draw up essential programs for preventing the consequences of this disease.

## OBJECTIVE

In this study, we aimed to measure the QoL of patients with b-TM using SF-36 for the first time in southern Iran. In addition, we wanted to determine predictive factors.

## MATERIALS AND METHODS

In this cross-sectional study, a total of 101 patients with SHB β-TM who were being registered at the Hematology Research Center of Shiraz University of Medical Sciences were randomly enrolled during 2009. All the patients were more than 12 years old and had been transfusion-dependent since before the age of two years. They had been referred to receive regular blood transfusions, to a referral hospital in Shiraz, Southern Iran. All of them were using deferoxamine as iron-chelating therapy, with poor or good compliance. The diagnosis had been made according to their hemoglobin electrophoresis result and complete blood count. The study protocol was approved by both the Institutional Review Board and the Ethics Committee of Shiraz University of Medical Science. Informed written consent was obtained from all patients.

The patients’ demographics, including age, sex, marital status, education and economical class, were recorded. The disease characteristics consisted of iron-chelating therapy status, splenectomy and presence of comorbid chronic diseases.

The Persian version of the SF-36 questionnaire was used in this study, and this had previously been translated from English and validated.^11^ The SF-36 is a well-recognized, self-administered QoL scoring system. It consists of eight independent scales and two major dimensions. The eight multi-item scales include physical functioning (PF), role-physical (RP), bodily pain (BP), general health (GH), vitality (VT), social functioning (SF), role-emotional (RE) and mental health (MH). The first five scales are summarized into the physical health dimension and the last three scales into the mental health dimension.^12^^,^^13^

It has previously been shown that the SF-36 questionnaire can be completed through an interview, by phone or by computerized administration, even though it is designed as a self-administered questionnaire.^14^ Thus, we used both self-administration and interviews for filling out the questionnaire. All participants were asked to fill out the form. Those who were illiterate were assisted by a trained researcher, who filled out the form for them at a face-to-face interview. The responses to the SF-36 questionnaire were scored quantitatively based on the answers to between 2 and 10 multiple choice questions, with a score of between 0 and 100, in accordance with the guidelines of the Clinical Outcome Evaluation System software.^12^^,^^13^

### Statistical analysis

Statistical analysis was done using the Statistical Package for Social Sciences software, version 17.0 (SPSS Inc., Chicago, IL, USA). The results were expressed as mean values ± standard deviations and proportions, as appropriate. We conducted univariate analysis to determine the correlations between clinical and demographic factors and the QoL score. The independent-samples t-test was used to compare scores between pairs of subgroups of patients relating to each of the variables of sex, age, splenectomy, marital status, comorbid diseases and iron chelation.

The analysis of variance (ANOVA) test and subsequently the least significant difference (LSD) test were used to make comparisons between the scores in different categories of patients relating to educational level and economic class. Multivariate analysis by means of multiple linear regression analysis was done to determine the predictive factors that independently influenced the QoL scores among thalassemic patients. Two-tailed P-values less than 0.05 were considered statistically significant.

## RESULTS

Overall, we included 101 patients with b-TM including 44 males (43.6%) and 57 females (56.4%) with a mean age of 19.5 ± 4.3 years (range 12-38). Table 1 demonstrates the patients’ demographic and clinical characteristics.

**Table 1.** Demographic and disease characteristics of 101 Iranian patients with SHB beta-thalassemia major

Variables	Values
Age (years) (mean ? SD)	19.5 ? 4.3
Sex	
Male	44 (43.6%)
Female	57 (56.4%)
Education	
Illiterate	4 (4%)
Junior School	27 (26.7%)
High School	23 (22.8%)
Bachelor of Science	14 (13.9%)
Master of Science	27 (26.7%)
Doctorate	6 (5.9%)
Economic class	
Low income	28 (27.7%)
Moderate income	45 (44.6%)
High income	28 (27.7%)
Marital status	
Single	95 (94.1%)
Married	6 (5.9%)
Splenectomy	
Yes	32 (31.7%)
No	69 (68.3%)
Comorbidities	
Cardiological disorders	10 (9.9%)
Diabetes mellitus	8 (7.9%)
Hepatitis B or C	5 (4.9%)
Others	5 (4.9%)
No comorbidity	73 (72.4%)
Iron-chelating therapy	
Good compliance	86 (85.1%)
Poor compliance	15 (14.9)

SD = standard deviation.

Table 2 shows the SF-36 score results on the eight scales, the two summary statuses for physical and mental health, and total scores for the male and female patients with b-TM. For the total score and the summaries of physical and mental health, there were no statistically significant differences between the male and female patients. Differences were only seen on two scales (BP and RE), such that the male patients showed significantly higher scores than the female patients (P = 0.041, P = 0.009 respectively).

**Table 2.** Health-related quality of life of the patients with beta-thalassemia major in southern Iran, according to the Short Form-36 (SF-36) score

	Total (n = 101)	Men (n = 44)	Women (n = 57)	P-value
Physical functioning	86.9 ? 12.9	88 ? 14	85 ? 12	0.334
Role-physical	65.4 ? 20.1	67 ? 20	64 ? 20	0.365
Bodily pain	63.7 ? 22.1	69 ? 21	60 ? 22	0.041^*^
General health	62.5 ? 22.8	64 ? 23	61 ? 23	0.472
Vitality	62.5 ? 22.1	66 ? 22	59 ? 22	0.104
Social functioning	69.5 ? 24.2	73 ? 25	67 ? 23	0.249
Role-emotional	70.8 ? 21.2	77 ? 20	66 ? 21	0.009^*^
Mental health	60.9 ? 20.4	65 ? 21	58 ? 20	0.091
Physical health	68.2 ? 15.7	71 ? 17	64 ? 15	0.102
Mental health	65.2 ? 18.3	69 ?19	62 ? 17	0.063
Total SF-36	67.8 ? 16.1	71 ? 17	65 ? 15	0.056

All values are presented as mean ? standard deviation. ^*^Statistically significant.

Table 3 demonstrates the results from the univariate analysis on covariates in relation to the physical health, mental health and total SF-36 scores. There were no statistically significant relationships between the scores and age, education, splenectomy or marital status (P > 0.05). The patients with higher incomes had significantly higher scores for physical health, mental health and total scores, in comparison with the patients with lower incomes (P < 0.001). Moreover, the patients with good compliance with iron-chelating therapy had higher scores than shown by the patients with poor compliance, in all of these three evaluated scores (P < 0.002). Lack of comorbidities was correlated with having a significantly better physical health score (P = 0.031).

**Table 3.** Univariate analysis on covariates associated with Short Form-36 (SF-36) scores among patients with beta-thalassemia major in southern Iran

	Physical health mean ? SD	P value	Mental health mean ? SD	P value	Total SF-36 score mean ? SD	P value
Age (years)						
? 20 (n = 69)	69.3 ? 15.9	0.288	66.9 ? 18.9	0.181	69.1 ? 16.5	0.281
³ 21 (n = 32)	65.8 ? 15.2		61.7 ? 16.6		65.2 ? 15.1	
Education level						
< High-school diploma (n = 31)	65.6 ? 15.6	0.088	62.1 ? 17.6	0.099	64.7 ? 16	0.104
High-school diploma (n = 23)	64.2 ? 16.3		61 ? 18.5		64.4 ? 16.3	
> High-school diploma (n = 47)	71.8 ? 14.9		69.4 ? 18.1		71.4 ? 15.5	
Economic level						
Low income (n = 28)	58.7 ? 14.6	< 0.001*	53.7 ? 16	< 0.001*	57.1 ? 14.4	< 0.001*
Moderate income (n = 45)	69.1 ? 14.4		66.5 ? 16.5		69.3 ? 14.2	
High income (n = 28)	76.4 ? 13.9		74.7 ? 17.8		76 ? 15.1	
Splenectomy						
Yes (n = 32)	65.4 ? 13.7	0.225	62.2 ? 16.6	0.251	65.1 ? 15.3	0.232
No (n = 69)	69.5 ? 16.4		66.7 ? 19		69.5 ? 17.3	
Compliance with iron-chelating therapy						
Good (n = 86)	70.3 ? 14.4	0.001*	67.8 ? 16.2	0.001*	69.8 ? 14.6	0.002*
Poor (n =15)	56.2 ? 17.5		50.8 ? 23.3		56.1 ? 19.5	
Marital status						
Single (n = 95)	68.2 ? 15.9	0.937	65.1 ? 18.5	0.779	67.7 ? 16.3	0.796
Married (n = 6)	68.2 ? 13.3		67 ? 15		69.6 ? 12.5	
Comorbidities						
No (n = 73)	70.3 ? 15.7	0.031*	67.1 ? 19.3	0.074	69.6 ? 16.6	0.073
Yes (n = 28)	62.8 ? 14.6		60.6 ? 14.6		63.1 ? 13.7	

*Statistically significant. SD = standard deviation.

In Table 4, we have summarized the results from multiple linear regression models for covariables relating to the SF-36 scores. The presence of comorbidities, poor compliance with iron-chelating therapy and lower income were inversely correlated with the score for the physical health dimension (b*-*coefficients = -0.197, -0.285 and -0.332; and P = 0.027, 0.002 and < 0.001, respectively). Furthermore, poor compliance with iron-chelating therapy and lower income were associated with lower scores for mental health and for the total SF-36 (Mental health: b*-*coefficients = -0.289 and -0.357; and P = 0.002 and P < 0.001, respectively; Total SF-36: b*-*coefficients = -0.236 and -0.383; and P = 0.004 and < 0.001, respectively).

**Table 4.** Multiple linear regression analysis on determinants of the Short Form-36 (SF-36) scores among patients with beta-thalassemia major in southern Iran

	R	R ^2^	F (DF; P value)	Beta coefficient	P value
Physical health	0.509	0.259	11.3 (3; < 0.001*)		
Comorbidities				-0.197	0.027*
Iron-chelating therapy				-0.285	0.002*
Economic level				-0.332	< 0.001*
Mental health	0.485	0.235	15.1 (2; < 0.001*)		
Compliance with iron-chelating therapy				-0.289	0.002*
Economic level				-0.357	< 0.001*
Total SF-36 score	0.489	0.239	15.2 (2; < 0.001*)		
Compliance with iron-chelating therapy				-0.236	0.004*
Economic level				-0.383	< 0.001*

*Statistically significant. DF = degrees of freedom. F = F test. R = correlation coefficient.

The independent variables were entered into the multiple linear regression models as follows: comorbidities (1 = yes); compliance with iron-chelating therapy (1 = poor); economic level: high and moderate economic levels were combined together as one group, thus producing two groups: low income and high or moderate income (1 = low income).

## DISCUSSION

In this study, the QoL of the patients with b-TM was assessed using SF-36. The SF-36 questionnaire is a well-known self-administered instrument for assessing QoL in the general population, as well as among patients with various diseases. The Persian-translated version of this questionnaire has been used to determine reliability and validity in different studies in normal populations,^15^^,^^16^ and also among b-thalassemic patients in Iran.^11^^,^^17^ We found worse physical health scores for the b-TM patients who had comorbidities, poor compliance with iron-chelating therapy or lower income. Poor compliance with iron-chelating therapy and low income were also found to be related to lower scores for the mental health dimension as well as for total SF-36.

In comparison with the results from a SF-36 health survey that was conducted on healthy Iranian individuals, our patients had lower mean scores in all scales except in PF.^15^ Similar to our study, Khani et al.^17^ reported decreased quality of life of patients with b-TM in North of Iran.

Beta-thalassemia major becomes apparent between the ages of 6 and 24 months.^18^ Therefore, homozygotes are involved with this disease since the early months of life. The difference between thalassemic children and their peers gives rise to feelings of anguish, self-scorn and many destructive senses, thereby resulting in decrease life expectancy.^19^ The QoL of b-thalassemic patients has increased due to medical advances, improvements in therapeutic methods, safer blood transfusion, new iron chelators and regular treatment methods.^20^^,^^21^ Stem cell and bone marrow transplantation and promotion of gene therapy are promising as well.^22^

Among the population studied, 68% were not more than 20 years old. We did not find any significant relationship between age and the QoL scores. Sobota et al.^23^ investigated QoL in a longitudinal thalassemia cohort aged over 14 years, compared with the norms in the United States, along with the influence of clinical factors. Differing from our results, they showed worse QoL among older patients. They also found lower scores among women. Similarly, in our study, women had lower scores on two scales (BP and RE), although the scores for the two main dimensions and total SF-36 did not show any significant differences between male and female patients. Older age and female gender are factors known to be correlated with lower QoL scores in the general population.^15^

Our study showed that 70% of the patients evaluated were at high-school diploma, bachelor’s degree or postgraduate degree level. However, educational level did not show any significant association with the SF-36 scores. This is in contrast with the results from Tajvar et al.,^24^ who found that education was a significant determinant of lower physical health-related quality of life in an elderly population.

Comorbidities were present in 28% of our participants, and these included cardiological disorders, diabetes mellitus, hepatitis B or C, and other related complications. The presence of these associated diseased states was an independent predictive factor for lower physical health summary scores. Consistently with our results, Sobota et al.^23^ reported lower scores among thalassemic patients who had higher numbers of these complications. Particular attention deserves to be paid towards decreasing these co-morbidities and complications through regular periodic follow-up, examination and appropriate management of disease complications in thalassemic patients.

Due to the inevitable associated problems in b-TM, such as cardiological disorders, diabetes mellitus, hepatitis, dependency on periodic blood transfusion and infertility, it is expected that only a few patients may be able to marry. In our study, only 6% of the patients had been married. Nevertheless, marital status had no significant relationship with the SF-36 score.

Patients presenting good compliance with iron-chelating therapy had higher QoL scores for the physical and mental health dimensions, as well as higher total scores. It is possible that patients who have good compliance with iron-chelating therapy have better attitudes toward their health, which results in better practices in relation to the disease-associated problems that they face. This differs from what was reported by Sobota et al.,^23^ who showed that there was no significant association between any compliance measurement and QoL, among 57 thalassemic patients who were on deferoxamine alone.

In our study, higher income was a significant determinant of higher QoL scores for the physical and mental health dimensions, as well as higher total scores. This was concordant with the results from Tajvar et al.,^24^ in a study on an elderly Iranian population. Obviously, with better economic status, different populations and especially thalassemic patients would be able to confront their disease-related problems more easily.

Our results support the impact of thalassemia major as a chronic disease on QoL similarly to other reports that have evaluated the impact of other chronic diseases on QoL by SF-36 questionnaire in Iranian populations. These chronic disease states include epilepsy,^25^ coronary artery disease^26^ rheumatoid arthritis,^27^ and diabetic foot ulcers.^28^^,^^29^

 To promote QoL among thalassemic patients, a variety of factors should be taken into account by health-related policymakers, with special attention to patients presenting any comorbid diseases, poor compliance with iron-chelating therapy and lower socioeconomic status. Psychosocial support should be scheduled for patients and their families in order to prevent mental health issues, behavioral problems and illness complications.^30^ Promotion of knowledge about the benefits of therapeutic and prophylactic processes may reduce the level of emotional distress and increase the quality of therapy.^31^ Attendance by social workers and psychologists at treatment centers, to provide support and regular peripheral interviews with patients, can improve QoL. Promotion of social knowledge, regular clinical checkups and modern treatment strategies could help to increase the hopes and life expectancy of thalassemic patients.^32^

Our study was limited by the lack of a control group, and therefore we were unable to compare the QoL scores of thalassemic patients with similar scores in a normal population. In addition, because this was a patient population in a single referral institution, the results may not be representative of patients in Iran with this condition.

## CONCLUSION

We found that the presence of disease complications, poor compliance with iron-chelating therapy and poor economic status were predictors of worse QoL among patients with b-TM. Prevention and proper management of disease-related complications and greater knowledge among patients regarding the importance of comorbidity management and good compliance with iron-chelating therapy, as well as consideration for these patients’ psychosocial and financial support, could be helpful in coping better with this chronic disease state.
